# Geographical and climatic risk factors of cutaneous leishmaniasis in the hyper-endemic focus of Bam County in southeast Iran

**DOI:** 10.3389/fpubh.2023.1236552

**Published:** 2023-11-09

**Authors:** Mohammad Amin Ghatee, Iraj Sharifi, Niloufar Mohammadi, Bahareh Esmaeili Moghaddam, Mohammad Hasan Kohansal

**Affiliations:** ^1^Department of Medical Microbiology, School of Medicine, Yasuj University of Medical Sciences, Yasuj, Iran; ^2^Leishmaniasis Research Center, Kerman University of Medical Sciences, Kerman, Iran; ^3^Student Research Committee, Yasuj University of Medical Sciences, Yasuj, Iran; ^4^Pathology and Stem Cell Research Center, Kerman University of Medical Sciences, Kerman, Iran; ^5^School of Medicine, Bam University of Medical Sciences, Bam, Iran; ^6^Department of Parasitology and Mycology, Tabriz University of Medical Sciences, Tabriz, Iran

**Keywords:** cutaneous leishmaniasis, geo-climatic, geographic information system, control program, Leishmania tropica

## Abstract

**Introduction:**

Cutaneous leishmaniasis (CL) is a prevalent debilitating disease in many countries, particularly in Iran, the Middle East, North Africa, and South America. Bam County is the most important highly endemic focus of anthropometric CL in Iran and has been under consideration by WHO. This study investigated the environmental and geographic factors affecting the occurrence and distribution of CL in this focus.

**Methods:**

Demographic data and the home addresses of CL patients diagnosed from 2015 to 2020 were retrieved from the Leishmaniasis Center of Bam in southeast Iran. The effects of mean annual rainfall (MAR), mean annual humidity (MAH), mean annual temperature (MAT), maximum annual temperature (MaxMAT), minimum annual temperature (MinMAT), mean annual humidity (MAH), mean annual evaporation (MAE), mean annual frosty days (MAFD), mean annual snowy hours (MASH), elevation, and land cover on the distribution of CL were analyzed using geographical information systems (GIS) and univariate and multivariate regression models.

**Results:**

Of 847 patients studied, 50.9% (*n* = 431) were female and 49.1% (*n* = 416) were male. The age classes 0–10 (*n* = 246) and 11–20 (*n* = 145) showed the highest frequency of patients, respectively. Leishmaniasis patients were reported from 66 villages/cities (11.8%) out of 561 residential areas in Bam County. Univariate analysis showed that urban settings (OR = 21.66), agriculture (OR = 5.73), orchards (OR = 5), salty land (OR = 1.05), and temperatures (OR = 2.37, 2.79 and 3.47) had positive effects on CL occurrence (*p < 0.05*), while altitude, precipitation, humidity, evaporation, and the number of frozen days had negative effects. Multivariate analysis identified urban settings (OR = 13.6), orchards (OR = 6.29), agriculture (OR = 5.82), and minimum temperature (OR = 2.38) as the most significant determinants of CL occurrence in this region.

**Conclusion:**

Environmental and ecological factors play an important role in the distribution of CL in Bam County. The high-risk zones for CL are cities/large villages, agricultural and orchard areas in lower altitudes and with warmer climates and lower rainfall and humidity. This model can guide researchers and health managers to properly conduct CL control programs and allocate budgets.

## 1. Introduction

Leishmaniasis is a vector-borne parasitic disease caused by a protozoan of the genus *Leishmania*. The disease has been classified by the World Health Organization (WHO) as one of the most important neglected tropical diseases due to its serious impacts on economic development and well-being (1). Several different forms of leishmaniasis have been reported which are mainly classified into three main categories, including cutaneous leishmaniasis (CL), visceral leishmaniasis (VL), and muco-cutaneous leishmaniasis (MCL). Leishmaniasis is transmitted by the female sandfly vector of the genus *Phlebotomus* (throughout Africa/Asia) or *Lutzomyia* (in America) and the clinical consequences of the disease range from self-limiting CL to fatal VL (2–5).

Leishmaniasis is endemic in 97 countries, with an estimated 350 million people at risk for the disease and an incidence of approximately 50,000 to 90,000 cases of VL and 0.7 to 1.2 million cases of CL (6–8). Iran is among the first six countries in the world challenged with the CL and is classified as a high-risk country for the disease (9) although VL also is endemic in some parts of country (10). The number of reported Leishmania cases in Iran decreased from 23,202 in 2008 to 13,124 in 2019 (11).

CL is generally classified into two forms based on clinical symptoms and epidemiological characteristics: zoonotic CL (ZCL) or rural CL due to Leishmania major, and anthroponotic CL (ACL) or urban CL due to Leishmania tropica (8, 12–14). ZCL is endemic in the northeastern, central, western, and southwestern provinces of Iran, with some foci in southeastern regions. The main vectors of ZCL are *Phlebotomus papatasi*, and the disease is transmitted to humans through desert rodents of the Gerbillidae family, including *Rhombomys opimus, Tatera indica, Meriones libycus, M. persicus, M. hurrianae*, and *Nesokia indica*. On the other hand, ACL is mostly reported from large cities and small towns and villages in Shiraz (southwest), Tehran (central), Mashhad (northeast), Kerman, and Bam (southeast), with some overlaps in some endemic foci in Iran. The primary vector of ACL is *Ph. sergenti*, which mainly transmits the disease among humans and possibly dogs (15, 16).

GIS is a powerful and efficient technology that has been used to investigate the spatial patterns of various public health problems, including parasitic diseases. Using spatial data and analyzing it can improve the health of society in a short time and be cost-effective (17–19). The epidemiology CL is related to interactions between the parasite, vector, host, and the environment. Recently, GIS-based studies have shown an association between the prevalence of CL in endemic regions and factors such as climatic and environmental situations, human travel and immigration, and socio-economic status (20, 21).

Several GIS-based studies have been conducted to analyze the risk factors associated with CL in Iran. Sharafi et al. showed an association between a high incidence of CL and areas with maximum temperature, mean of temperature, mean of evaporation, sunny days, and wind velocity (22). Holakouie-Naieni et al. fund that CL most prominently occurs in regions with dry and desert climates (23). Ghatee et al. study identified land cover, slope, elevation, and close proximity to cattle sheds as the most effective factors in CL occurrence (18).

Bam County in southeast Iran has been considered by the Iranian Health Ministry and World Health Organization as the most important ACL focus of Iran (24, 25) with a spectrum of land covers where a destroying earthquake occurred in 2003. About 40,000 CL cases have been reported in a half-century period with the highest incidence in the nearby years after earthquake in 2003 and a decreasing trend in the recent years (26). DNA based molecular methods revealed *L. tropica* in almost all human CL cases while no *L. major* infection was reported (27). Also 2.6% of trapped *Ph. sergenti* was infected by *L. tropica* in Bam area (28).

Although different epidemiological and molecular studies on CL have been carried out in Bam County, no research has been conducted to analyze CL distribution based on the environmental and climatic variables by GIS technology in in this area. The current study updated CL data from this highly endemic focus and investigated the distribution and occurrence of CL based on the geo-climatic factors in this hyper-endemic focus of CL in Southeast Iran using GIS.

## 2. Methods

### 2.1. Study areas

**Figure 1:**
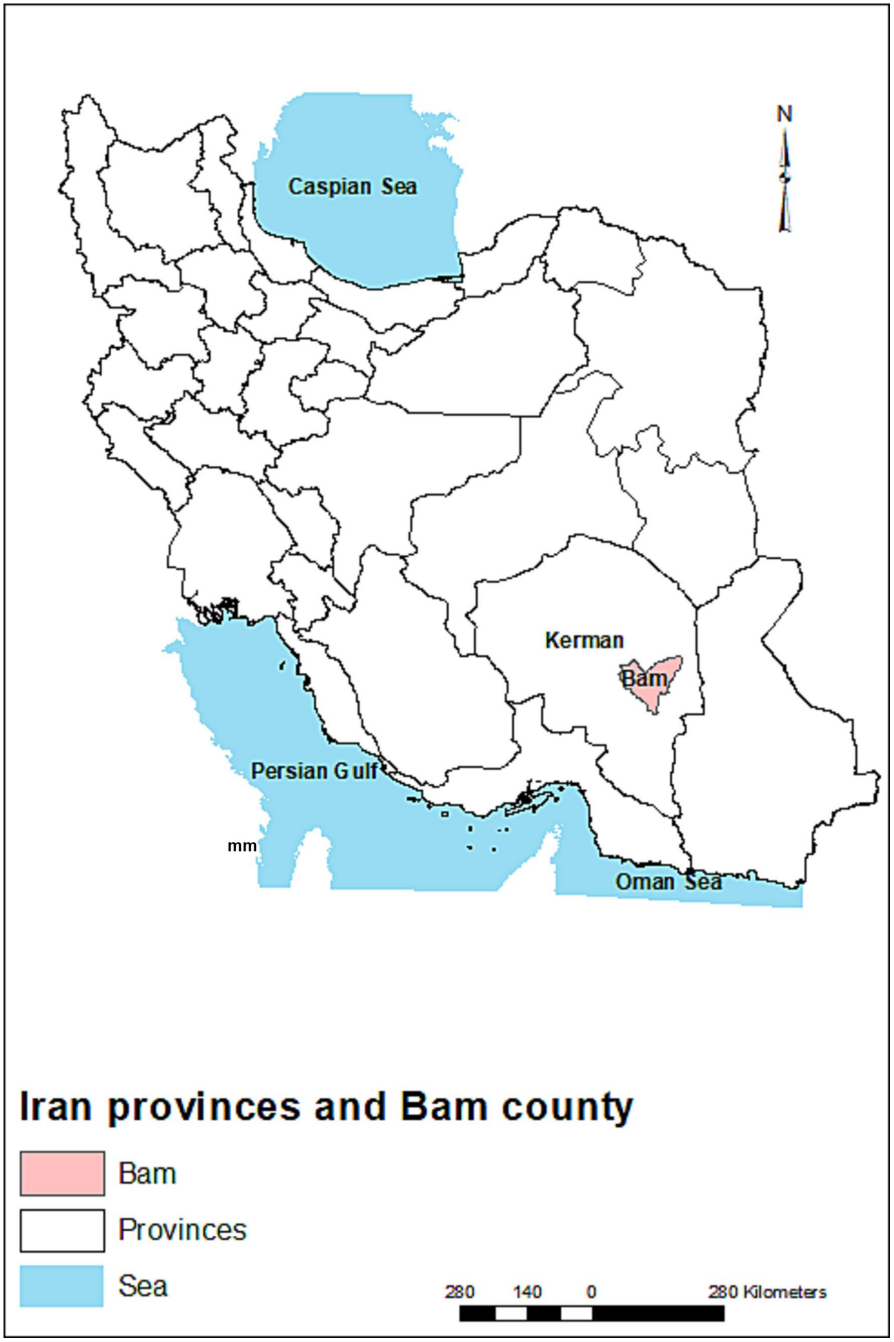
The location of Bam County in Kerman province in southeast Iran.

Kerman is the largest province of Iran, with a population of 2.7 million. The province covers an area of 181,714 km^2^ in the southeastern part of the country. Bam is one of the historical cities and counties in the Kerman province (Figure 1) with a population of ~318,241 people. This city has a warm and arid climate and is located in geographical coordinates of 29°6′ N and 58°21′ E and an elevation of 1,050 m (29). The main agricultural and orchard products of this county are wheat, barley, alfalfa, palm, various types of citrus, and pistachio.

### 2.2. Data collection

CL cases were diagnosed based on the epidemiological, clinical, and historical evidence and results of microscopic examination (e.g., Giemsa/Leishman-stained skin scraping) and confirmed by the physicians at the Leishmaniasis Center. Home addresses and demographic data of all patients from 2015 to 2020 were obtained from patients’ medical records at the Bam *Leishmania* Center. The validity of home addresses was checked by calling each patient using the phone number obtained from their medical records.

### 2.3. Geospatial data

The residences of CL patients were located on the point shapefile layer of Bam County, including villages and cities. From 2015 to 2020, data on mean temperature, minimum temperature, maximum temperature, rainfall, humidity, evaporation, number of frozen days, and number of sunny hours were acquired from 12 synoptic metrological stations located in Kerman Province including Kerman, Bam, Sirjan, Shahr-e-Babak, Baft, Rafsanjan, Anar, Kahnouj, Zarand, Shahdad, Lalehzar and Jiroft cities. These stations collected meteorological data at 6 or 3-h intervals throughout the day and night. The data were obtained from the Kerman Province Weather Bureau.

Mean annual temperature (MAT), maximum annual temperature (MaxMAT), minimum annual temperature (MinMAT), mean annual humidity (MAH), mean annual evaporation (MAE), mean annual frozen days (MAFD), and mean annual sunny hours (MASH) were calculated based on the meteorological data. After testing different interpolation methods, iso-thermal, iso-hydrate, iso-evaporation, frozen days, and sunny hour’s raster layers were generated using inverse distance weighted (IDW) and iso-humid was generated by tension-based spline method with a 1 × 1 km for Kerman Province.

#### 2.3.1. Geo-climatic analysis

ArcGIS version 10.5^1^ was used to analyze geo-climatic data. The Bam County layers were clipped from various raster and vector layers which were generated for the same surface of Kerman Province. Bam County’s villages and cities point shapefile layers were extracted with the raster layers. The geometric intersection of the layer acquired from the extraction of all raster layers with land cover (polygonal) vector layers were computed by the identity tool to make the final layer in which each point represented properties of all the overlapped identity features from the above-mentioned raster and vector layers. The attribute of this layer was converted to an Excel format for statistical analysis.

### 2.4. Statistical analysis

Having described the spatial distribution and demographic characteristics of CL patients, the effect of climatic and environmental factors on the occurrence of CL was assessed. Accordingly, geo-climatic data of CL-infected and non-infected points were analyzed using univariate and multivariate logistic regression (forward stepwise method) models. The statistical analyzes were performed using SPSS version 21.

## 3. Results

A total of 847 patients’ data were retrieved from the Leishmaniasis Center of Bam. Of these, 50.9% (*n* = 431) and 49.1% (*n* = 416) were female and male, respectively. The age groups with the highest frequency of patients were 0–10 (29.04%) and 11–20 (17.11%), respectively.

### 3.1. Univariate analysis

Leishmaniasis patients were reported from 66 villages/cities (11.8%) of 561 residential areas in Bam County. MAT (*p* = 0.002, OR = 2.795) MaxMAT (*p* = 0.004, OR = 3.478), and MinMAT (*p* = 0.001, OR = 2.378) were effective factors in the occurrence of CL. An increase of one centigrade degree of MAT, MaxMAT, and MinMAT increased the chance of CL by 279, 347, and 237%, respectively (Table 1).

**Table 1:** Univariate analysis of the effects of climatic factors on CL in Bam County.

Variable^a^	*p* value	OR	CI	
MAT	0.002	2.795	1.464	5.336
MaxMAT	0.004	3.478	1.501	8.06
MinMAT	0.001	2.378	1.438	3.932
MAR	<0.001	0.826	0.761	0.897
MAH	0.003	0.93	0.887	0.976
MAE	<0.001	0.983	0.976	0.99
MAFD	<0.001	0.885	0.832	0.942
MASH	0.73	1.007	0.967	1.049
DEM	<0.001	0.999	0.999	1
Poor range (constant)	0			
Moderate range	0.919	0.93	0.233	3.709
Agriculture	0.005	5.731	1.713	19.172
Orchard	0.031	5	1.163	21.5
Low forest and Woodland	0.998	0	0	
Salt land	0.043	5.909	1.055	33.104
Urban	0.002	21.667	3.007	156.141
Bare land	0.825	1.204	0.233	6.208

a The unit of temperatures, rain, humidity, evaporation, frozen day, and sunny hours were centigrade degrees, millimeters in the year, percent, a millimeter in month, day, and hour.

MAT, mean annual temperature; MaxMAT, maximum annual temperature; MinMAT, minimum annual temperature; MAR, mean annual rainfall; MAH, mean annual humidity; MAE, mean annual evaporation; MAFD, mean annual frosty days; MASH, mean annual snowy hours.

**Figure 2:**
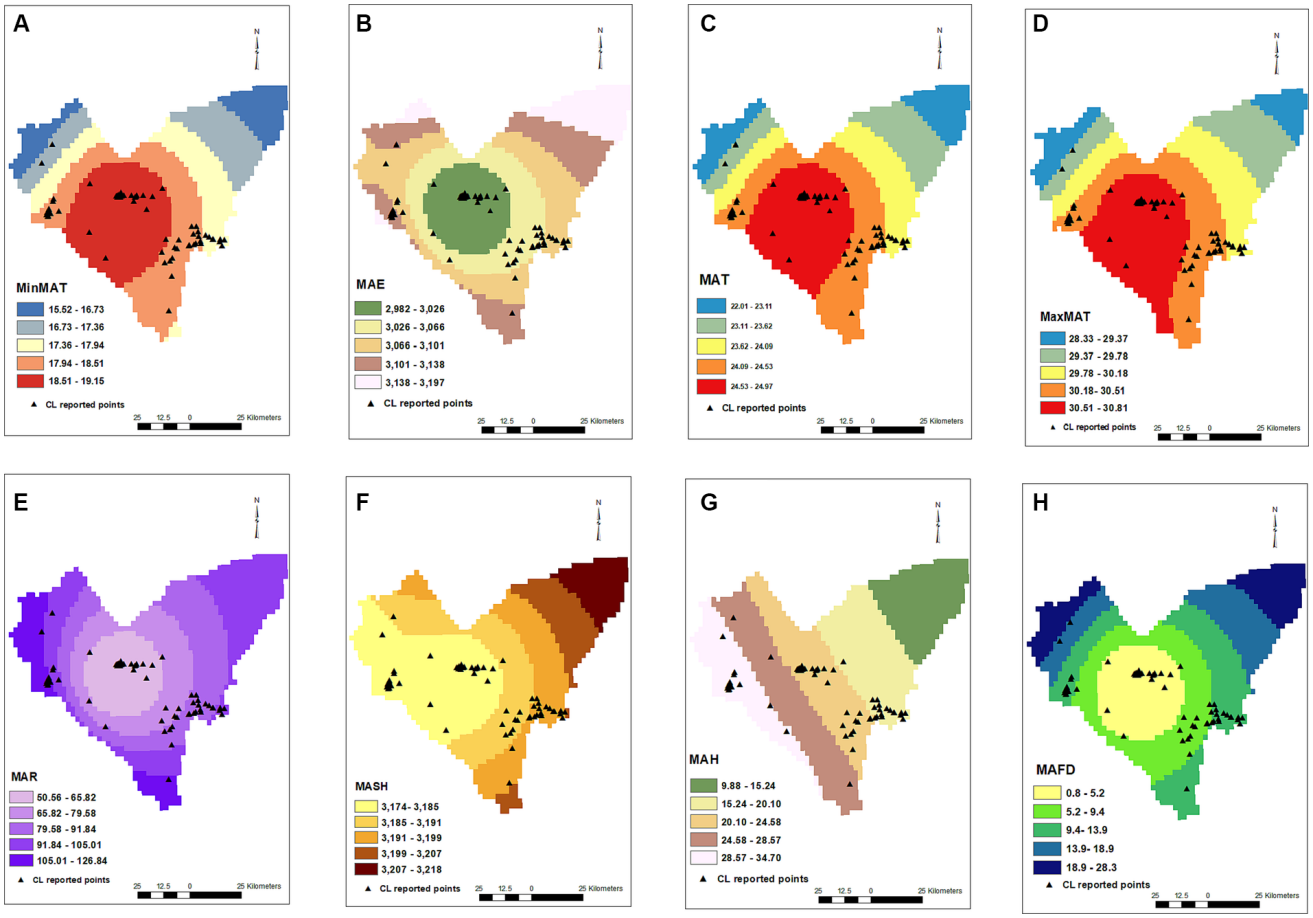
Minimum annual temperature **(A)**, mean annual evaporation **(B)**, mean annual temperature **(C)**, maximum annual temperature **(D)**, mean annual rainfall **(E)**, mean annual snowy hours **(F)**, mean annual humidity **(G)**, mean annual frosty days **(H)** maps and distribution of CL infected points.

MAR (*p* = < 0.001, OR = 0.826), MAH (*p* = 0.003, OR = 0.93), and MAE (*p* = < 0.001, OR = 0.983) were also effective on the CL distribution in the studied areas, decreasing the chance of diseases by 18, 7, and 2%, respectively, for an increase of each unit of mentioned variables. MAFD (*p* = < 0.001, OR = 0.885) and MASH (*p* = 0.73, OR = 1.007) were other studied factors on the occurrence of CL in the Bam County, where an increase in each frozen day decreased the chance of CL by 12%. MASH did not show an effect on CL in the current study (Figure 2).

**Figure 3:**
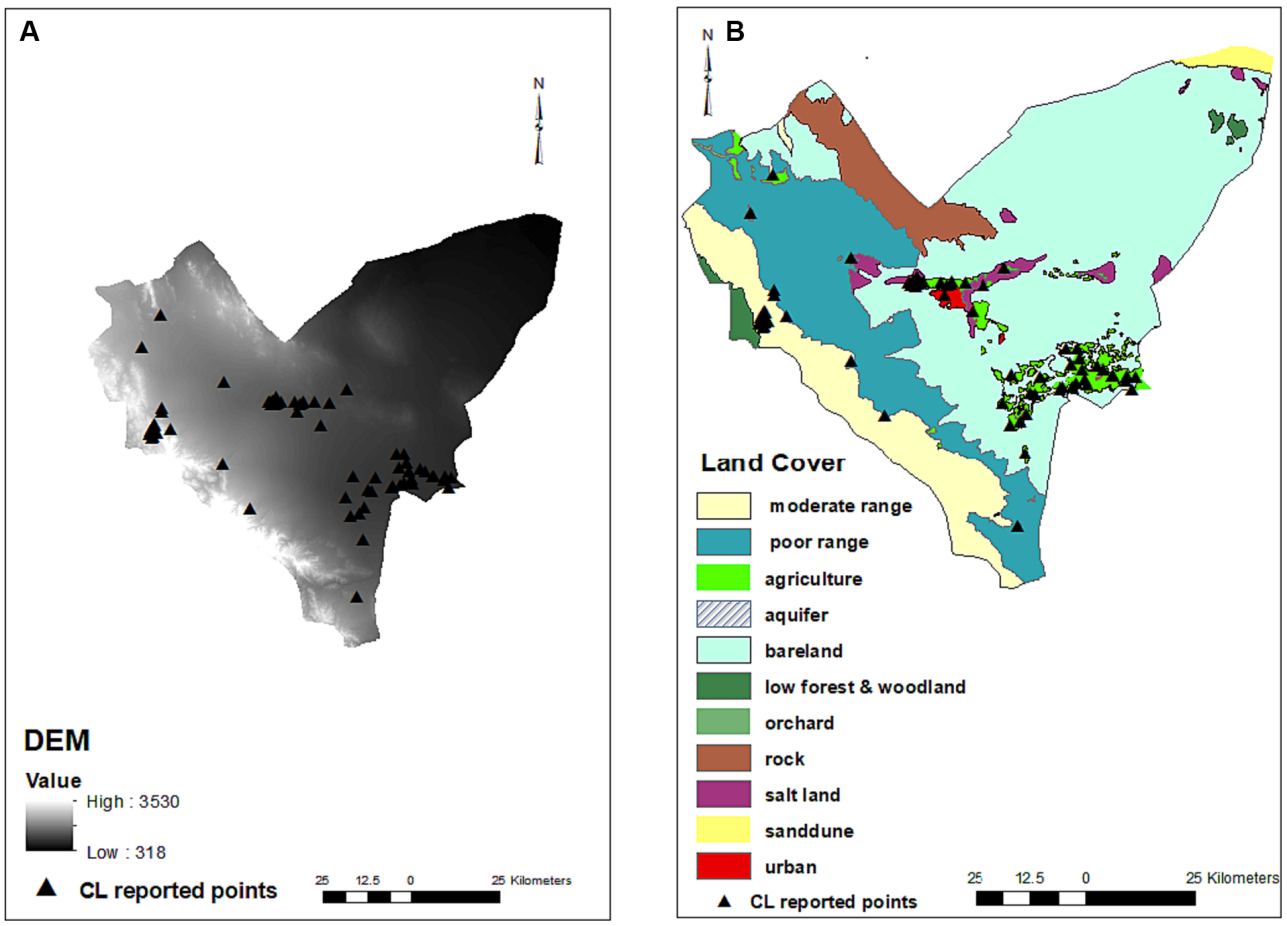
Digital elevation model **(A)** and land covers **(B)** maps and distribution of infected points. No residential places were on rock and aquifer areas (There are 561 residential places including cities and villages in Bam County that cannot be shown on the map due to crowding and so only those had CL patients were present on the map). Most of CL infected points were found on the urban settings (red), agriculture, orchards (green) and parts of salt lands (purple) where are in close proximity of agriculture areas. No infected points were observed on the salt lands on the east of county.

Among the different land types covering the studied area, agriculture (*p* = 0.005, OR = 5.731), orchard (*p* = 0.031, OR = 5), salt land (*p* = 0.043, OR = 5.909), and urban settings (*p* = 0.002, OR = 21.667) were shown to be effective on the CL occurrence, increasing the chance of the disease by 5.7, 5, 5.9 and 21.6 times, respectively. Moderate rangeland (*p* = 0.919), low forest and woodland (*p* = 0.998), and bare land (*p* = 0.825) did not affect the occurrence of disease. Elevation (DEM) negatively affected the occurrence of CL (*p* < 0.001, OR = 0.999) (Table 1; Figure 3).

### 3.2. Multivariate analysis

A forward stepwise multivariate logistic regression model was designed for the variables that were significantly effective on the occurrence of CL in the univariate analysis, including MAT, MaxMAT, MinMAT, MAR, MAH, MAE, MAFD, DEM, and land covers to assess the concomitant effect of independent factors. In the final step, MinMAT and land cover were included in the model and other factors did not show significant effect and were automatically removed from the model. MinMAT (*p* = 0.001, OR = 2.382), and land covers of agriculture (*p* = 0.005, OR = 5.822), orchard (*p* = 0.015, OR = 6.296), and urban (*p* = 0.011, OR = 13.606) were the effective factors in the final multivariate model (Table 2).

**Table 2:** Multivariate analysis of geo-climatic factors associated with CL in Bam County.

Variable	*p* value	OR	CI	
MinMAT	0.001	2.382	1.399	4.056
Poor range (constant)	0	–	–	–
Moderate range	0.8	0.835	0.208	3.353
Agriculture	0.005	5.822	1.726	19.635
Orchard	0.015	6.296	1.435	27.627
Low forest and Woodland	0.998	0	0	–
Salt land	0.166	3.469	0.597	20.168
Urban	0.011	13.606	1.816	101.935
Bare land	0.734	1.133	0.256	6.936

MinMAT, minimum annual temperature.

## 4. Discussion

Urban settings, orchard areas, agriculture areas, and MinMAT had the greatest effect on the occurrence of CL in the Bam County, southeast Iran. Temperatures, rain, humidity, evaporation, frozen days, elevation, and salt land area were determined to be factors in the occurrence of CL when assessed independently from other factors.

Urban setting land cover was shown to have the greatest effect on the occurrence of CL in the current study. Urban settings are large residential areas including cities and large villages that are identified in the primary satellite images and then drawn as a part of the land cover maps. Different studies from various areas in Iran have shown a significant increase in the risk of leishmaniasis in large and more populated residential areas (17, 18, 30). The dominant type of CL in Bam County is ACL caused by *L. tropica*, which is known as urban leishmaniasis (8, 31) The main vector of ACL in Bam County is *Ph. sergenti* (77%) which prefers living indoors and is in close contact with humans. In Bam County, 85 and 81% of Phlebotomus species caught from indoor human and animal places were *Ph. sergenti* (32). Moreover, there are a notable number of orchards, including palm and citrus trees around homes in Bam city and other cities and villages in this area, which may provide shade, moisture, and suitable breeding places for sandflies (33). A systematic review in 2020 concluded that although during the first half of the twentieth century, living in areas away from population centers or proximity to forests were important risk factors for leishmaniasis, over time, urban and peri-urban dwellers were at the greatest risk of leishmaniasis.

Vector adaptation to urban areas and the expansion of these areas to surrounding vegetation, and proximity to agricultural areas as natural breeding grounds for vectors and probable reservoirs, especially in developing countries, increase the chances of acquiring leishmaniasis (20). Additionally, the high density of population in urban areas resulting from population migration from rural areas to urban areas for work and residence due to the severe drought in Iran in recent decades (34) increases the probability of CL transmission in urban settings.

Results of this study showed that orchards and agriculture areas had a notable effect on the occurrence of CL in the Bam County. These lands provide suitable circumstances for the growth of vectors. Sand flies as *Leishmania* vectors prefer shadowy humid habitats like orchards and agriculture areas for the laying of eggs, survival, and development of immature stages and those who work there are at a higher risk of being bitten (35).

In Bam County, also, there are cultivating areas in neighboring cities and villages and a notable number of trees are in the courts of homes. Therefore, the abundance of sand fly population close to human settlements can increase the risk of disease. The result of our study was consistent with other studies that reported a notable number of leishmaniasis patients to work or have been on agricultural farms (36–39). However, the investigation conducted by Mokhtari et al. (40) in Ilam demonstrated that the development of irrigation for agricultural aims in an arid area significantly decreased ZCL cases. They inferred that the nest of rodents is a place for sandflies to rest, and irrigation of fields can cause destruction of these places and eventually suggested that the development of irrigated agriculture might work as a preventive factor for CL prevalence.

In Bam, almost all cases of leishmaniasis are ACL caused by *L. tropica* which circulates mainly among humans while rodents do not play a role in its circle. Accordingly, another study with an ACL-dominant focus in Afghanistan showed the effect of irrigated farming and close proximity to the Harrirod river on the increase of ACL cases (41).

Salt land was found as another effective land cover that increases the chance of CL in the Bam County. There are a few studies that have investigated the effect of salt land on leishmaniasis though no association was found (17, 42). Observation of the land cover map shows that CL was reported from parts of salt lands that were just nearby to agriculture areas while there is no infected place in other parts of salt lands especially in the eastern region of Bam County (Figure 3). Therefore, we assume that the effect found for this land type belonged to the neighboring agricultural area although salty regions were found as effective land cover for some other parasitological diseases (43). Some studies have reported that behavioral activities and the life cycle of sand flies and reservoir hosts of leishmaniasis are impacted by changes in climatic factors of rainfall, humidity, and atmospheric temperature (44–46). We observed a positive effect of temperatures on the CL occurrence that was in line with studies conducted in other areas with close climatic conditions in Iran. Hanafi-Bojd et al. (35) showed that mean annual temperature was among the most important factors on the presence of CL vectors especially in central Iran. Shirzadi et al. in a study that included provinces in the east and northeast of Iran showed a significantly positive relationship between CL incidence and land surface temperature (47). Mozafari et al. (48) reported that the maximum temperature of the warmest month and mean temperature of the warmest and driest quarters have the highest contribution to the distribution of the disease in the Golestan province. Various studies have confirmed that higher maximum temperature, lower rainfall, and lower relative humidity are effective factors in the geographic distribution of *leishmaniasis*, especially in semi-arid areas of central Iran (47, 49, 50). Also, in arid areas of eastern Iran and another focus in southeast Iran, mainly including semi-arid areas, lower rainfall increased the chance of disease (17, 18). In the current study, higher temperatures, lower humidity, rainfall, and the lower number of frozen days had a positive effect on the occurrence of CL. Bam County, in southeast Iran, is a part of the vast arid and semi-arid southeast, east-northeast, and central region of Iran. The lack of dependence on rainfall and weather humidity in the aforementioned studies is probably due to the reliance on underground water to support life in this arid region where sand flies are likely to exist on the limited humidity provided by water pulled from underground sources used for different purposes, including daily consumption, agriculture, animal husbandry, and industry.

Considering that rainfall has a correlation with elevation, where an increase in elevation and a consequent decrease in temperature lead to increased precipitation, there is a negative association between CL and elevation in the Bam County. This can be explained by the negative effect of coldness and precipitation on the survival of sandflies in high elevations. Galvez et al. (51) showed in a study in Spain that there was a decrease of 5–7% in the density of sandflies with each millimeter increase in rainfall.

Evaporation had a negative impact on CL in the current study. This may be explained by the limited humidity provided by confined water reservoirs and irrigated farms and gardens, which is vital for sandflies survival. Higher evaporation decreases humidity for sandflies in this semi-arid area. In contrast, in greener regions with high rainfall and humidity and a diversity of water resources in north Iran, higher evaporation was positively associated with CL (52). Generally, CL is more prevalent in low-humidity areas and therefore, higher evaporation may create such conditions in areas with higher humidity as well.

The main limitation of this study is the probable absence of some patients. Even though related data from all patients referred to Leishmaniasis Center in Bam during the studied period were included, some people may try to treat their disease by traditional methods and do not refer to the Leishmaniasis Center and therefore their data were not included here.

Implementation of active case-finding approaches could help assess the actual burden of the disease for planning future control programs. In the second place, due to some cofounding factors including socio-cultural determinants and movement of people between various areas, it was not feasible to provide a decisive conclusion for a comprehensive CL control program.

## 5. Conclusion

Urban setting, orchard and agriculture areas, and MinMAT were the most important determinants of the distribution of CL in the region of Bam in southeastern Iran. Areas including cities/large villages, agricultural and orchards, which are also located in lower altitudes and have a warmer climate and lower rainfall and humidity, constitute the main risk zone for CL in Bam County, the most important ACL focus of Iran. The current data showed the higher risk zone of CL in this area and can be used by health and financial authorities for directed allocation of the leishmaniasis control budget and prediction of new foci of CL in neighboring regions. Current data also can be used to identify areas where have so far not been reported as infected but have the potential to become disease hotspot in any possible future epidemic.

## Data availability statement

The raw data supporting the conclusions of this article will be made available by the authors, without undue reservation.

## Ethics statement

The studies involving humans were approved by Ethics Committee of Bam University of Medical Sciences (IR.MUBAM.REC.1399.020). The studies were conducted in accordance with the local legislation and institutional requirements. The participants provided their written informed consent to participate in this study.

## Author contributions

MG, MK, and IS designed the study. MK, BM, and NM collected the data. MG and MK performed analyzes and interpreted the results and supervised the conduct of this study. All authors contributed to the article and approved the submitted version.

## Footnotes

^1^ http://www.esri.com/arcgis
